# Supplementary material for: Androgens Modulate the Immune Profile in a Mouse Model of Polycystic Ovary Syndrome
Source: Adv Sci (Weinh). 2024 May 20;11(28):2401772. doi: 10.1002/advs.202401772 (PMC11267352; doi:10.1002/advs.202401772)
Supplement: Supplementary file 1 — Supporting Information [file ADVS-11-2401772-s001.pdf]

ADVANCED SCIENCE
Open Access

Supporting Information

for *Adv. Sci.*, DOI 10.1002/advs.202401772

Androgens Modulate the Immune Profile in a Mouse Model of Polycystic Ovary Syndrome

*Sara Torstensson, Angelo Ascani, Sanjiv Risal, Haojiang Lu, Allan Zhao, Alexander Espinosa, Eva Lindgren, Maria H. Johansson, Gustaw Eriksson, Maya Barakat, Mikael C.I. Karlsson, Camilla Svensson, Anna Benrick and Elisabet Stener-Victorin**

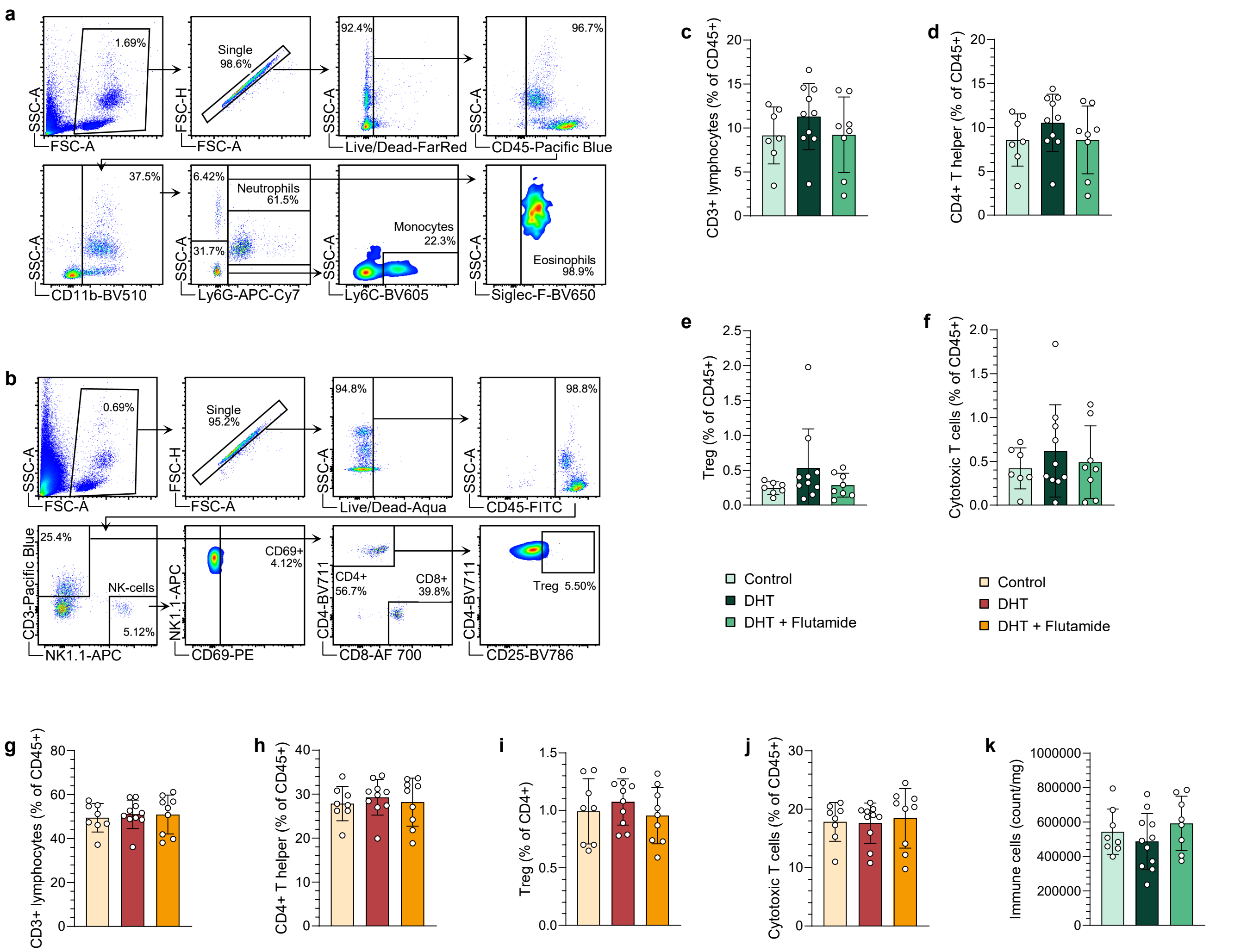

Supplementary figure 1. Immune populations in blood and secondary lymphoid organs are differently affected by androgen exposure. (**a**) Gating strategy myeloid cells in blood. (**b**) Gating strategy NK- and T cells in blood. (**c**) Frequency of $CD3^{+}$ T cells in spleen (*n* = 7 Control, 10 DHT, 8 DHT + Flutamide). (**d**) Frequency of $CD4^{+}$ T helper cells in spleen (*n* = 7, 10, 8). (**e**) Frequency of $CD4^{+}$ $CD25^{+}$ T reg cells in spleen (*n* = 7, 10, 8). (**f**) Frequency of $CD8^{+}$ cytotoxic T cells in spleen (*n* = 7, 10, 8). (**g**) Frequency of $CD3^{+}$ T cells in lymph nodes (*n* = 8, 10, 9). (**h**) Frequency of $CD4^{+}$ T helper cells in lymph nodes (*n* = 8, 10, 9). (**i**) Frequency of $CD4^{+}$ $CD25^{+}$ T reg cells in lymph nodes (*n* = 8, 10, 9). (**j**) Frequency of $CD8^{+}$ cytotoxic T cells in lymph nodes (*n* = 8, 10, 9). (**k**) Immune cell count in spleen (*n* = 8, 11, 8). Data are presented as means ± SD. n indicates the number of biologically independent samples examined. Statistical analysis was assessed by one-way ANOVA with Dunnett's multiple comparison (**c**-**d**, **f**-**i**, **k**) or by Kruskal-Wallis with Dunn's multiple comparison (**e**, **j**) Source data are provided as a Source Data File.

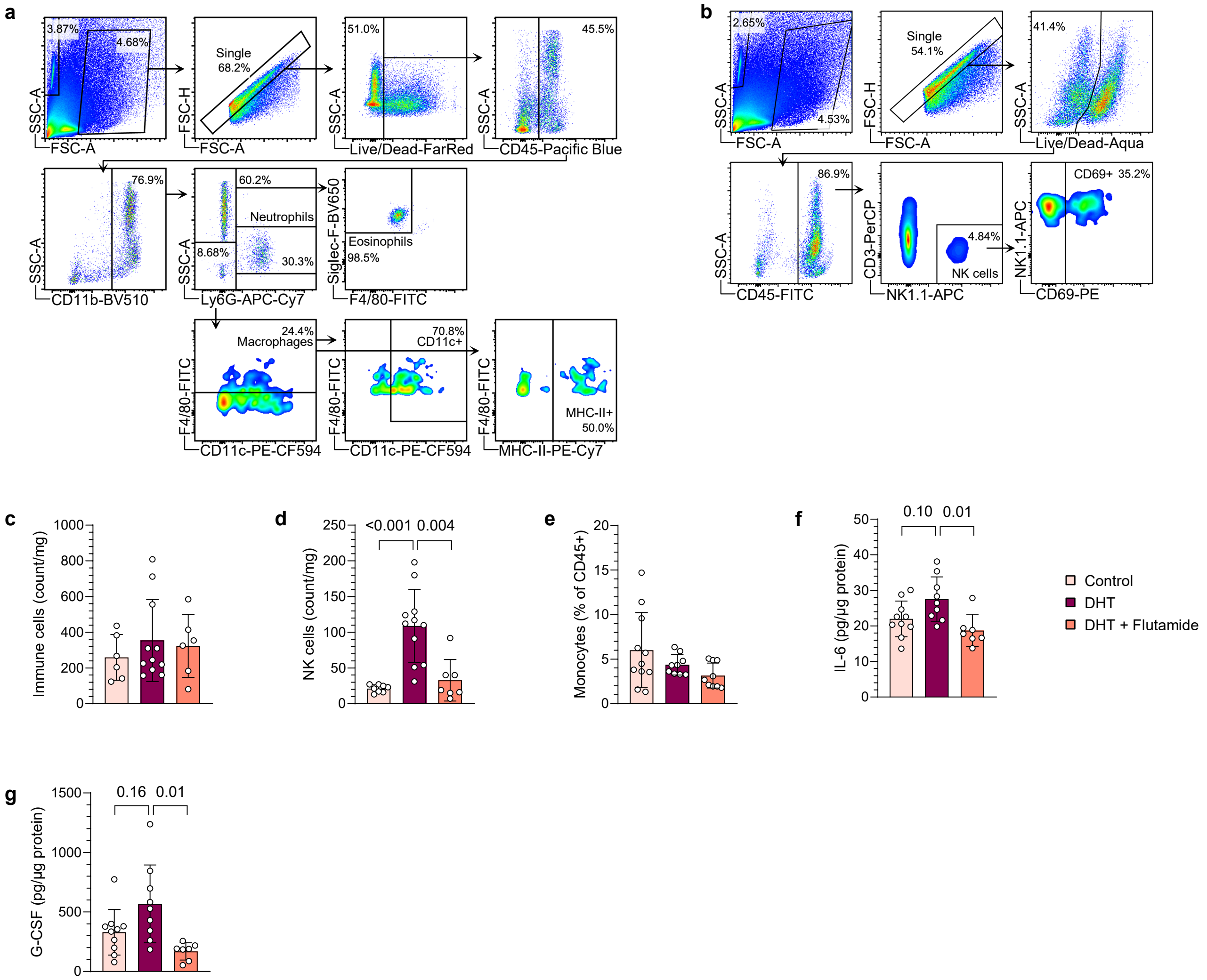

Supplementary figure 2. Uterine eosinophil and NK cell populations are markedly altered by androgen exposure in PCOS-like mice. (**a**) Gating strategy myeloid cells. (**b**) Gating strategy NK cells in uterus. (**c**) Number of CD45+ immune cells in uterus *(n* = 6 Control, 11 DHT, 6 DHT + Flutamide). (**d**) NK cell count uterus *(n* = 8, 11, 7). (**e**) Frequency of monocytes in uterus *(n* = 11, 9, 9). (**f**) IL-6 levels in uterus *(n* = 10, 9, 7). (**g**) Granulocyte-colony stimulating factor (G-CSF) levels in uterus *(n* = 10, 9, 7). Data are presented as means ± SD. n indicates the number of biologically independent samples examined. Statistical analysis was assessed by Kruskal-Wallis with Dunn's multiple comparison (**c-e**), or mixed-effects ANOVA with Bonferroni's multiple comparison test (**f-g**) and significant differences were indicated with p values. Source data are provided as a Source Data File.

Analyte	Comparison	95,00% CI	Summary	Adjusted P Value
IL-1a	DHT vs. Control	-2,54 to 29,3	ns	0,11
	DHT vs. Flutamide	-15,1 to 36,4	ns	0,58
IL-1b	DHT vs. Control	-0,961 to 3,48	ns	0,36
	DHT vs. Flutamide	-2,80 to 4,00	ns	>0,99
IL-2	DHT vs. Control	-2,03 to 45,3	ns	0,08
	DHT vs. Flutamide	-12,0 to 48,3	ns	0,29
IL-3	DHT vs. Control	-1,77 to 2,60	ns	>0,99
	DHT vs. Flutamide	-1,53 to 2,97	ns	0,86
IL-4	DHT vs. Control	-1,76 to 4,23	ns	0,64
	DHT vs. Flutamide	-1,50 to 4,23	ns	0,49
IL-9	DHT vs. Control	-2,71 to 33,6	ns	0,1
	DHT vs. Flutamide	-5,92 to 38,1	ns	0,17
IL-10	DHT vs. Control	-13,6 to 20,8	ns	>0,99
	DHT vs. Flutamide	-20,0 to 23,7	ns	>0,99
IL-12p40	DHT vs. Control	-6,13 to 65,4	ns	0,11
	DHT vs. Flutamide	-34,2 to 70,4	ns	0,79
IL-12p70	DHT vs. Control	-34,9 to 133	ns	0,34
	DHT vs. Flutamide	-27,3 to 136	ns	0,23
IL-13	DHT vs. Control	-109 to 199	ns	0,95
	DHT vs. Flutamide	-77,8 to 240	ns	0,42
IL-17a	DHT vs. Control	-2,57 to 3,91	ns	>0,99
	DHT vs. Flutamide	-4,44 to 5,54	ns	>0,99
GM-CSF	DHT vs. Control	-12,3 to 29,4	ns	0,65
	DHT vs. Flutamide	-13,0 to 27,1	ns	0,78
KC	DHT vs. Control	-7,85 to 32,5	ns	0,3
	DHT vs. Flutamide	-23,2 to 35,1	ns	>0,99
MIP-1a	DHT vs. Control	-9,91 to 2,35	ns	0,3
	DHT vs. Flutamide	-11,6 to 1,33	ns	0,13
MIP-1b	DHT vs. Control	-97,3 to 12,4	ns	0,15
	DHT vs. Flutamide	-67,2 to 14,3	ns	0,25
RANTES	DHT vs. Control	-198 to 370	ns	0,91
	DHT vs. Flutamide	-108 to 458	ns	0,26

Supplementary table 1. Cytokine levels in uterus (*n* = 10 Control, 9 DHT, 7 DHT + Flutamide). Data are presented as 95% confidence intervals (CI) with adjusted p-values. Statistical analysis was assessed by mixed-effects ANOVA with Bonferroni's multiple comparison test. Source data are provided as a Source Data File. ns indicates non-significant.

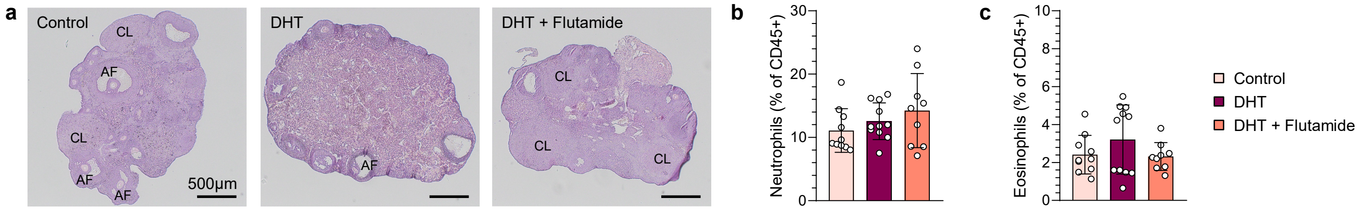

Supplementary figure 3. Ovarian macrophage populations are decreased by androgen exposure in PCOS-like mice. (**a**) Representative images of ovarian sections stained with H&E. Corpus lutea (CL) and antral follicles (AF) indicated. (**b**) Frequency of neutrophils in ovaries, expressed as percent of $CD45^+$ immune cells *(n* = 10 Control, 11 DHT, 9 DHT + Flutamide). (**c**) Frequency of eosinophils in ovaries *(n* = 10, 11, 9). Data are presented as means ± SD. n indicates the number of biologically independent samples examined. Statistical analysis was assessed by Kruskal-Wallis with Dunn's multiple comparison. Source data are provided as a Source Data File.

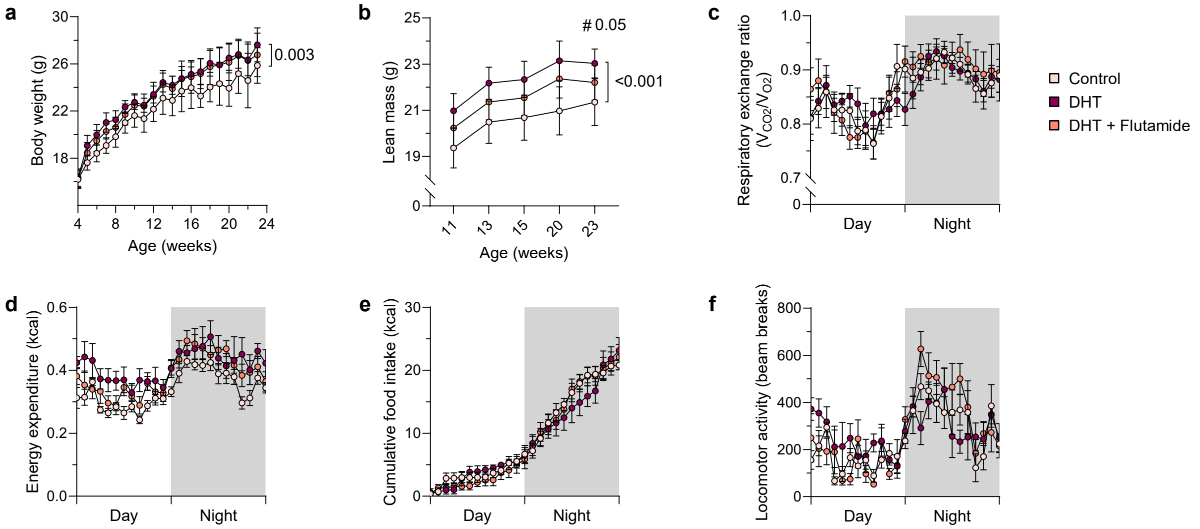

Supplementary figure 4. The peripubertal DHT-induced mouse model is a non-obese but insulin resistant model of PCOS. (**a**) Body weight (*n* = 12 Control, 11 DHT, 8 DHT + Flutamide). (**b**) Lean mass (*n* = 12, 11, 8). (**c**) Respiratory exchange ratio (*n* = 6, 5, 5). (**d**) Energy expenditure (*n* = 6, 5, 5). (**e**) Food intake (*n* = 6, 5, 5). (**f**) Locomotor activity (*n* = 6, 5, 5). Data are presented as means ± SD (**a-b**) and means ± SEM (**c-f**). # indicates statistical significance between DHT and DHT + Flutamide. n indicates the number of biologically independent samples examined. Statistical analysis was assessed by mixed-effects ANOVA with Bonferroni's multiple comparison test (**a**), two-way ANOVA with Bonferroni's multiple comparison test (**b**), or ANCOVA with body mass as covariate (**c-f**) and significant differences were indicated with p values. Source data are provided as a Source Data File.

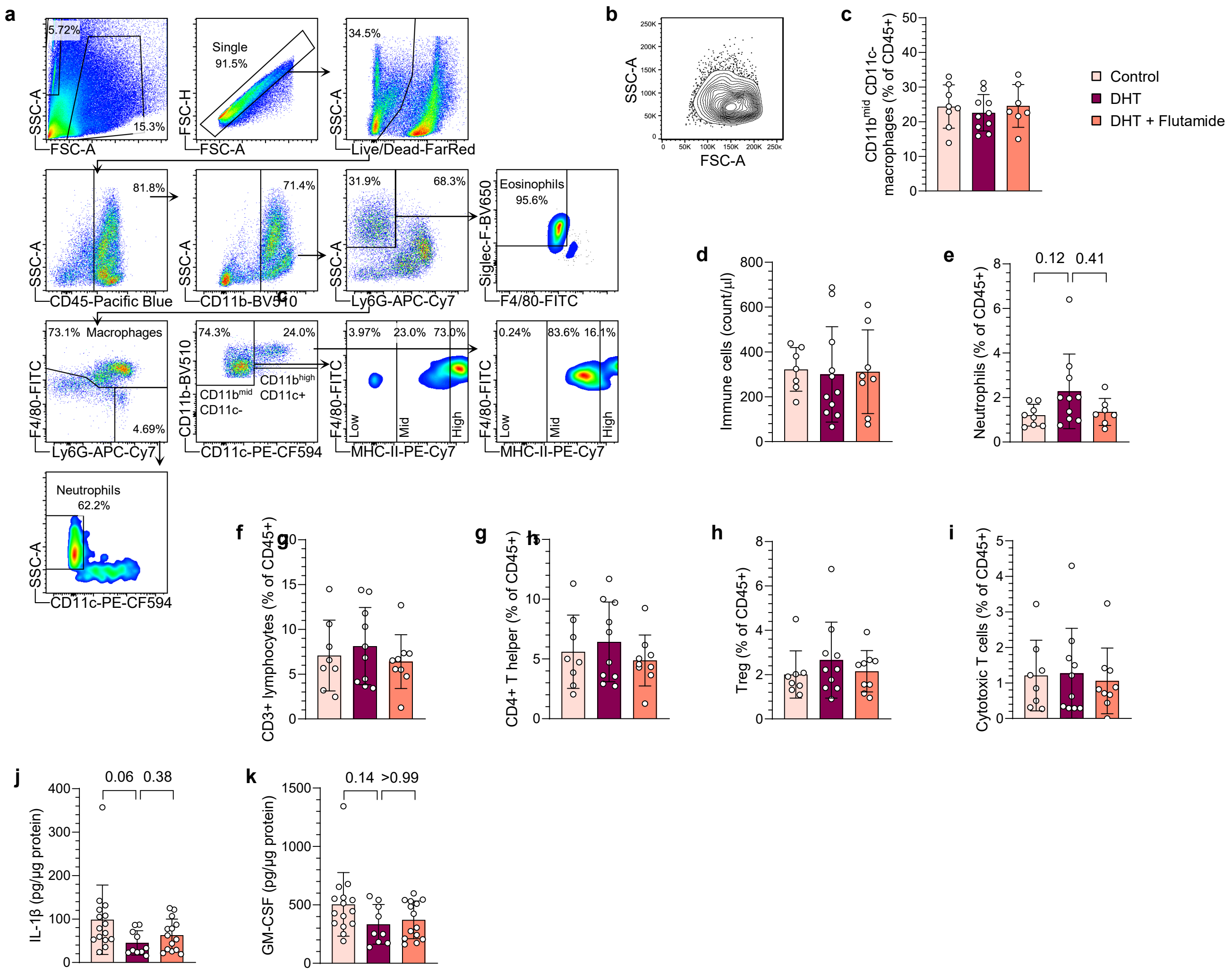

Supplementary figure 5. DHT-exposed PCOS-like mice display an aberrant immune profile in VAT albeit unaltered fat mass. (**a**) Gating strategy for myeloid cells in VAT. (**b**) Representative plot of macrophages in VAT. (**c**) Frequency CD11b^{mid}CD11c^{-} macrophages in VAT (*n* = 8 Control, 10 DHT, 7 DHT + Flutamide). (**d**) Number of CD45+ immune cells in VAT (*n* = 7, 11, 8). (**e**) Frequency of neutrophils in VAT (*n* = 8, 10, 7). (**f**) Frequency of CD3^{+} T cells in VAT (*n* = 8, 10, 9). (**g**) Frequency of CD4^{+} T helper cells in VAT (*n* = 8, 10, 9). (**h**) Frequency of CD4^{+} CD25^{+} T reg cells in VAT (*n* = 8, 10, 9). (**i**) Frequency of CD8^{+} cytotoxic T cells in VAT (*n* = 8, 10, 9). (**j**) IL-1β levels in VAT (*n* = 15, 10, 14). (**k**) GM-CSF levels in VAT (*n* = 15, 9, 14). Data are presented as means ± SD. n indicates the number of biologically independent samples examined. Statistical analysis was assessed by one-way ANOVA with Dunnett's multiple comparison (**c**-**d**, **f**-**g**), Kruskal-Wallis with Dunn's multiple comparison (**e**, **h**-**i**), or mixed-effects ANOVA with Bonferroni's multiple comparison test (**j**-**k**) and significant differences were indicated with p values. Source data are provided as a Source Data File.
